# Recurrent Lower-Extremity Purpura Mistaken for Cellulitis: Hepatitis C Virus-Associated Type III Mixed Cryoglobulinemic Vasculitis

**DOI:** 10.7759/cureus.113306

**Published:** 2026-07-24

**Authors:** Eric Walker, Chuckwumeka Ogbu

**Affiliations:** 1 Internal Medicine, Cape Fear Valley Medical Center, Fayetteville, USA

**Keywords:** cryoglobulinemia, cryoglobulinemic vasculitis, cutaneous small-vessel vasculitis, hcv-associated type iii mixed cryoglobulinemic vasculitis, palpable purpura

## Abstract

Mixed cryoglobulinemia is a small-vessel vasculitis mediated by cold-precipitable immune complexes, and chronic hepatitis C virus (HCV) infection is its most common cause. Cutaneous disease, classically palpable purpura of the lower limbs, is the most frequent manifestation but is readily mistaken for cellulitis or other dermatoses, delaying diagnosis. We describe a 61-year-old woman with chronic HCV, a long smoking history, and active cocaine use who experienced more than a year of recurrent, painful, pruritic erythematous - purpuric eruptions of the lower extremities that were repeatedly treated empirically as cellulitis without benefit. She also reported new easy bruising and skin fragility. Inflammatory markers were elevated, an extended antinuclear antibody panel was negative, and serum cryoglobulins were positive; immunofixation of the cryoprecipitate demonstrated type III (polyclonal) mixed cryoglobulinemia. Urinalysis showed hematuria with low-grade proteinuria, indicating renal involvement, and complement C4 was at the low end of normal. The findings established HCV-associated type III mixed cryoglobulinemic vasculitis. Cocaine history required consideration of levamisole-adulterated cocaine vasculopathy, an important mimic. She was started on corticosteroids with rheumatology co-management and HCV-directed direct-acting antiviral therapy as the cornerstone of treatment. Following completion of antiviral therapy and achievement of sustained virologic response, cutaneous vasculitis resolved, urinalysis normalized with no evidence of active glomerulonephritis, and the corticosteroid dose was successfully tapered. The patient remains on prednisone 5 mg daily with ongoing taper. This case underscores cryoglobulinemic vasculitis as a cause of recurrent, cellulitis-like, lower limb eruptions, and the primacy of antiviral cure in achieving vasculitis remission.

## Introduction

Cryoglobulins are immunoglobulins that reversibly precipitate at temperatures below 37°C and redissolve on rewarming [1]. According to the Brouet classification, type I consists of a single monoclonal immunoglobulin and is linked to lymphoproliferative disorders, whereas types II and III constitute mixed cryoglobulinemia: type II combines a monoclonal IgM with rheumatoid factor activity and polyclonal IgG, and type III contains only polyclonal immunoglobulins [1]. Mixed cryoglobulins can deposit in small vessels and trigger an immune complex-mediated vasculitis affecting skin, joints, peripheral nerves, and kidneys [2,3]. Chronic HCV infection is identified in the large majority of patients with mixed cryoglobulinemia and is regarded as the principal etiologic driver [3,4]. Almost a quarter of patients with mixed cryoglobulinemia have nephropathy at the time of their diagnosis, and renal involvement follows the diagnosis of cryoglobulinemia after a mean of 2.6-4 years [5,6]. Late diagnosis can allow for the progression of complications such as membranoproliferative glomerulonephritis and renal failure [6].

Cutaneous involvement is the most common feature, and the prototypical lesion is palpable purpura over the dependent lower extremities; weakness and arthralgia complete the classic clinical triad [2]. Because the eruption is erythematous, sometimes warm, and may be accompanied by edema, it is frequently misinterpreted as cellulitis, particularly when unilateral, leading to repeated antibiotic courses and diagnostic delay [7]. We present a patient whose recurrent lower-limb purpura was managed as cellulitis for over a year before serologic testing revealed HCV-associated type III mixed cryoglobulinemic vasculitis, and we discuss the diagnostic approach, key mimics including levamisole-adulterated cocaine vasculopathy, and the clinical course following HCV-directed therapy.

## Case presentation

History

A 61-year-old woman presented to a continuity clinic with recurrent left, then bilateral, lower-extremity eruptions. The lesions were red, intensely itchy and burning, associated with swelling, and accompanied by sharp pain radiating proximally. A similar episode approximately one year earlier had prompted exclusion of gout and an empiric antibiotic course with cephalexin 500 mg twice daily for 10 days for presumed cellulitis, without benefit; that rash later resolved spontaneously. At the index recurrence, she again received an antibiotic course for possible cellulitis. Over the following months, she also reported new, easy bruising with skin tearing after trivial trauma.

Her history included chronic HCV infection, chronic obstructive pulmonary disease (COPD), primary hypertension, chronic left knee and ankle pain, depression, and anxiety. Substance use included tobacco (approximately 23 pack-years) and cocaine. Outpatient medications included inhaled bronchodilator/corticosteroid therapy, gabapentin, amitriptyline, amlodipine, a proton-pump inhibitor, and as-needed analgesics.

Examination

Initially, she presented with an erythematous, macular, papular rash on the anterior right ankle (Figure 1).

**Figure 1:**
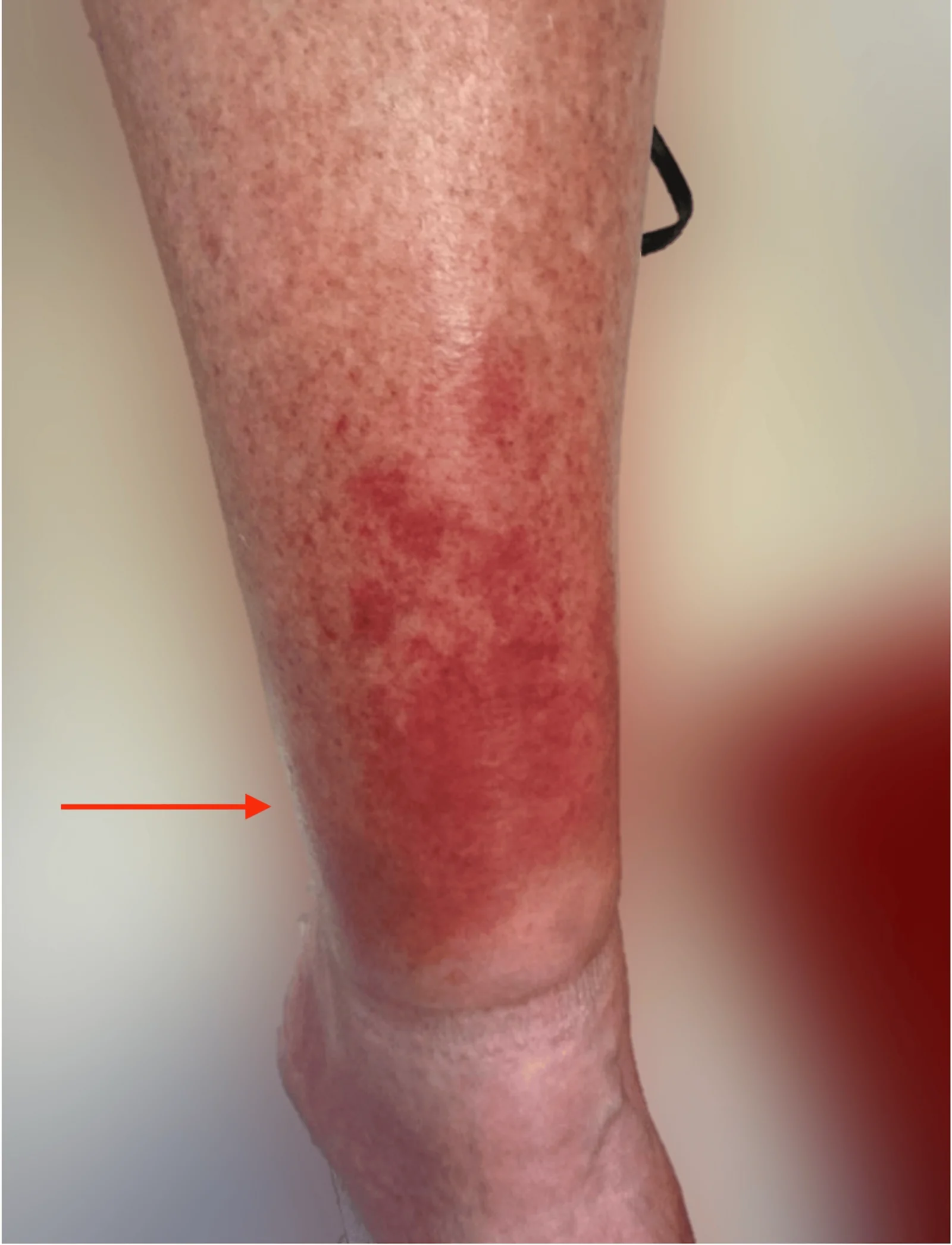
Right ankle demonstrating lower extremity edema with arrow pointing to erythematous-to-violaceous, partly confluent, maculopapular rash.

On follow-up, the left lower limbs showed erythematous-to-violaceous, partly confluent macular and faintly papular lesions over the distal leg, ankle, and foot (Figures 2, 3). The patient would later develop bilateral lower extremity edema, bruising, and right ankle tenderness. The remainder of the examination was notable for an obese, chronically ill-appearing woman without acute distress. Cardiopulmonary examination was unremarkable at rest.

**Figure 2:**
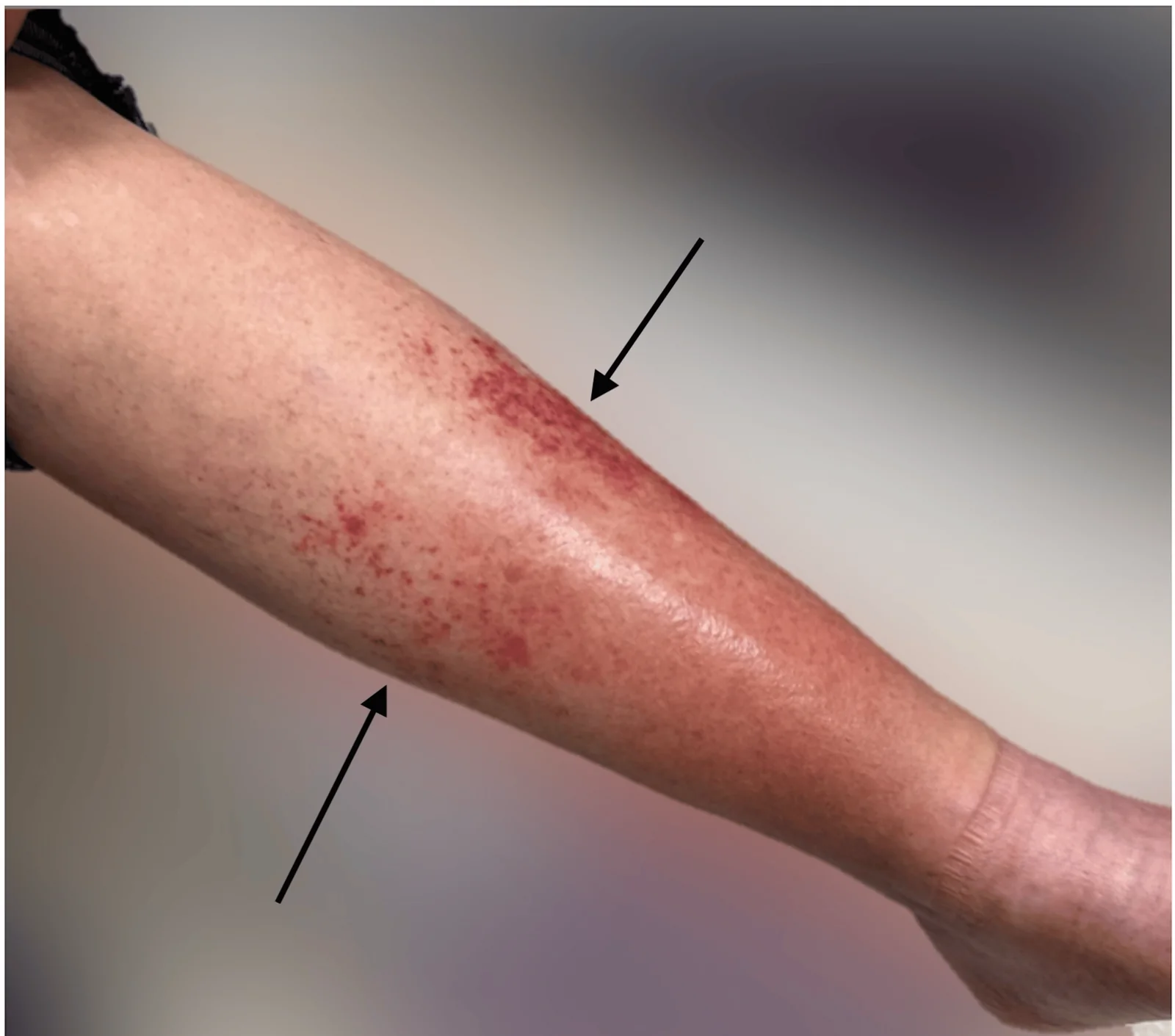
Left lower extremity demonstrating erythematous, macular, and papular lesions over the mid-distal anterior calf. Left and right arrows point to erythematous, maculopapular rash on the left and right sides of the lower extremity, respectively.

**Figure 3:**
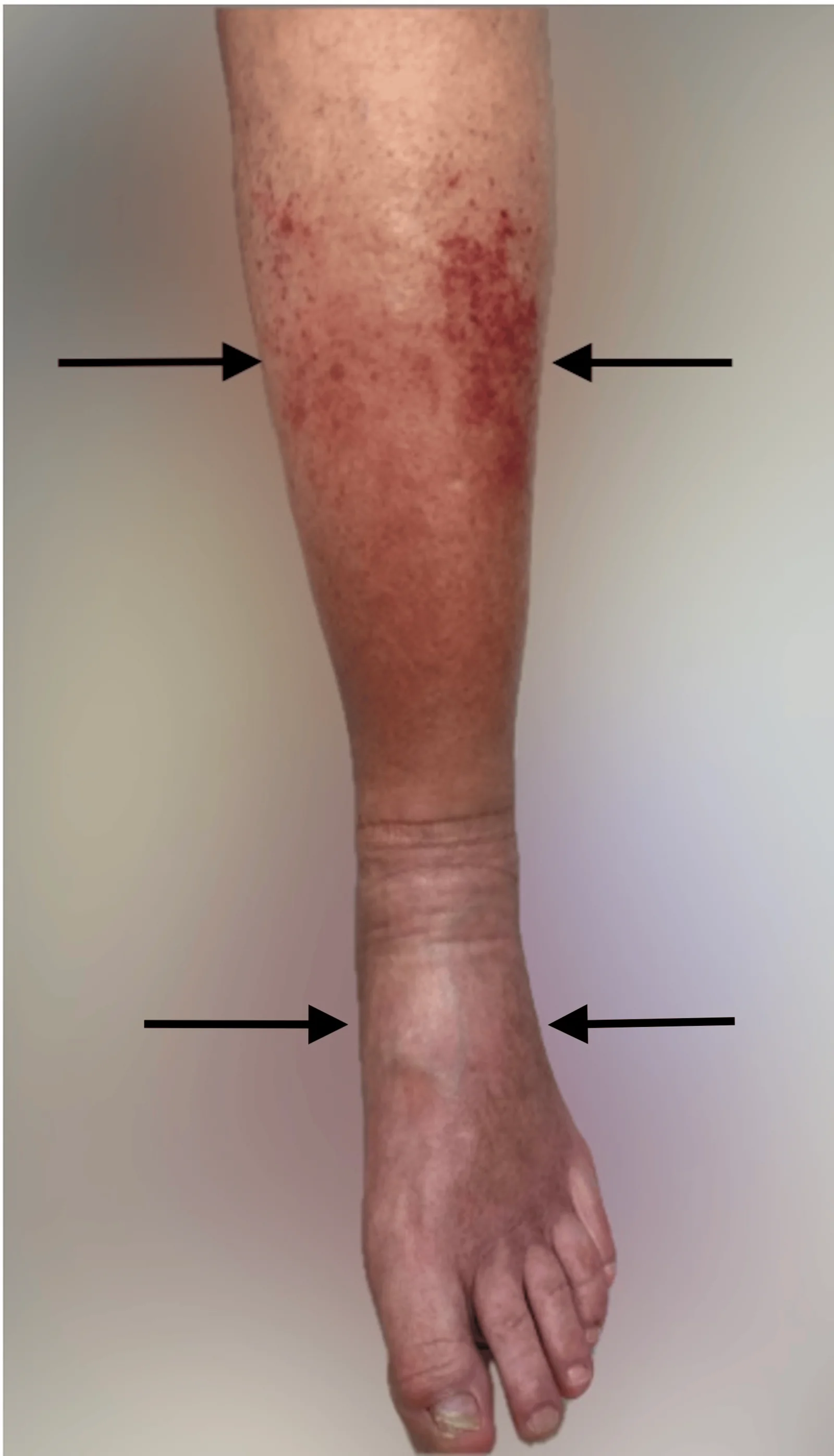
Clinical image of the left lower extremity. Upper arrows show macular and papular lesions over the distal calf and ankle. Lower arrows demonstrate violet discoloration and soft-tissue swelling of the left foot.

Laboratory studies

Inflammatory markers were elevated, with a C-reactive protein that rose on serial testing and a persistently elevated erythrocyte sedimentation rate. Serum cryoglobulins were qualitatively positive, and immunofixation of the cryoprecipitate demonstrated type III mixed cryoglobulinemia (polyclonal immunoglobulins); the cryoglobulin fraction was low (approximately 1%). Complement C3 was normal, and C4 was at the low end of normal. An extended antinuclear antibody panel was negative, with equivocal double-stranded DNA antibodies and negative extractable nuclear antigens, arguing against systemic lupus erythematosus or another defined connective tissue disease. Urinalysis showed hematuria and pyuria with low-grade proteinuria, and the spot urine protein-to-creatinine ratio was mildly elevated, indicating renal involvement. The hepatic panel showed mildly elevated alkaline phosphatase with previously elevated transaminases, HCV PCR 618,000 IU/mL, and HCV log10 5.791 log10 IU/mL, consistent with chronic HCV. Hepatitis C genotyping showed genotype 3 with METAVIR stage F2 hepatitis C. A urine drug screen was intermittently positive for cocaine (and at times fentanyl and benzodiazepines). Key laboratory results for the patient are reported in Table 1.

**Table 1:** Key laboratory findings †ANA panel included dsDNA (equivocal), RNP, Smith, SS-A/Ro, SS-B/La, Scl-70, Jo-1, chromatin, and centromere B; all negative. ‡Cocaine positive on previous screens; most recent screen also positive for fentanyl and benzodiazepines. Significant abnormal findings are placed in bold. H: above reference range; L: below reference range; HCV: hepatitis C virus; RT: reverse transcription; PCR: polymerase chain reaction; eGFR: estimated glomerular filtration rate; AST: aspartate aminotransferase; ALT: alanine aminotransferase; ANA: antinuclear antibody

Test	Value	Reference range
Inflammatory markers		
C-reactive protein	15 mg/L (H)	<5 mg/L
Erythrocyte sedimentation rate	41 mm/hr (H)	0-20 mm/hr
Cryoglobulins and complement		
Serum cryoglobulin, qualitative	Positive	Negative
Cryoprecipitate immunofixation	Type III (polyclonal)	-
Cryoglobulin fraction (cryocrit)	1.0% (H)	<0.5%
Complement C3	109 mg/dL	82-167 mg/dL
Complement C4	18 mg/dL (low-normal)	16-48 mg/dL
Viral serology		
HCV RT-PCR HCV log 10	5.791 log IU/mL	Undetected
Hepatitis C quantitation	618,000 IU/mL	Undetected
Hepatitis C virus antibody	Reactive	Non-reactive
Hepatitis A virus, IgM	Negative	Negative
Hepatitis B core, IgM	Negative	Negative
Hepatitis B surface antigen	Negative	Negative
Autoimmune serology		
ANA comprehensive panel†	Negative	Negative
Hepatic panel		
AST (peak 70 H, 2025)	13 U/L	13-39 U/L
ALT (peak 66 H, 2025)	6 U/L (L)	7-52 U/L
Alkaline phosphatase	139 U/L (H)	30-105 U/L
Albumin	3.7 g/dL	3.5-5.7 g/dL
Renal		
Creatinine	0.74 mg/dL	0.60-1.30 mg/dL
eGFR	>60 mL/min/1.73 m²	>60 mL/min/1.73 m²
Urinalysis	Hematuria, pyuria, trace protein	Negative
Urine protein/creatinine ratio	131 mg/g (H)	<150 mg/g
Hematology		
Hemoglobin	13.8 g/dL	12.0-16.0 g/dL
White blood cells	6.7 ×10³/µL	4.5-12.5 ×10³/µL
Platelets	309 ×10³/µL	150-450 ×10³/µL
Toxicology		
Urine drug screen	Cocaine positive‡	Negative

Imaging and other studies

Lower-extremity venous duplex ultrasonography was negative for deep vein thrombosis, excluding DVT as a cause of the unilateral swelling. Screening mammography was negative. Incidentally, cross-sectional imaging identified an approximately 2.6-3.1 cm enhancing, hypermetabolic mass at the mediastinal border of the right middle lobe (maximum standardized uptake value 7.9 on positron-emission tomography), with a small left pleural effusion, pleural thickening, healing left rib fractures, and cardiomegaly for which CT-guided biopsy was recommended (Figures 4, 5).

**Figure 4:**
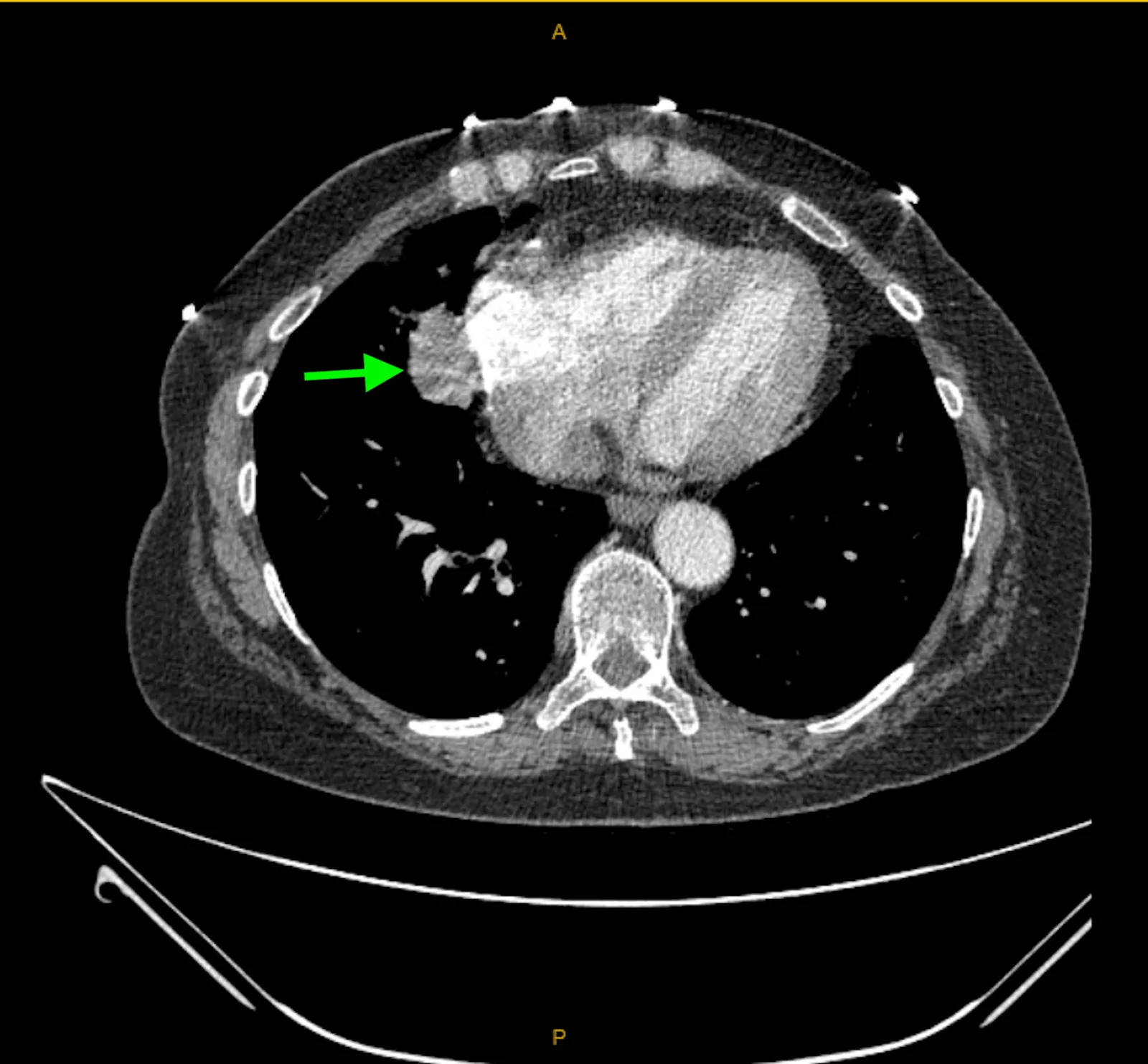
Incidental thoracic mass. Contrast-enhanced axial CT chest demonstrating a solid enhancing mass (green arrow points to the mass) at the mediastinal border of the right middle lobe adjacent to the right atrium. CT: computed tomography

**Figure 5:**
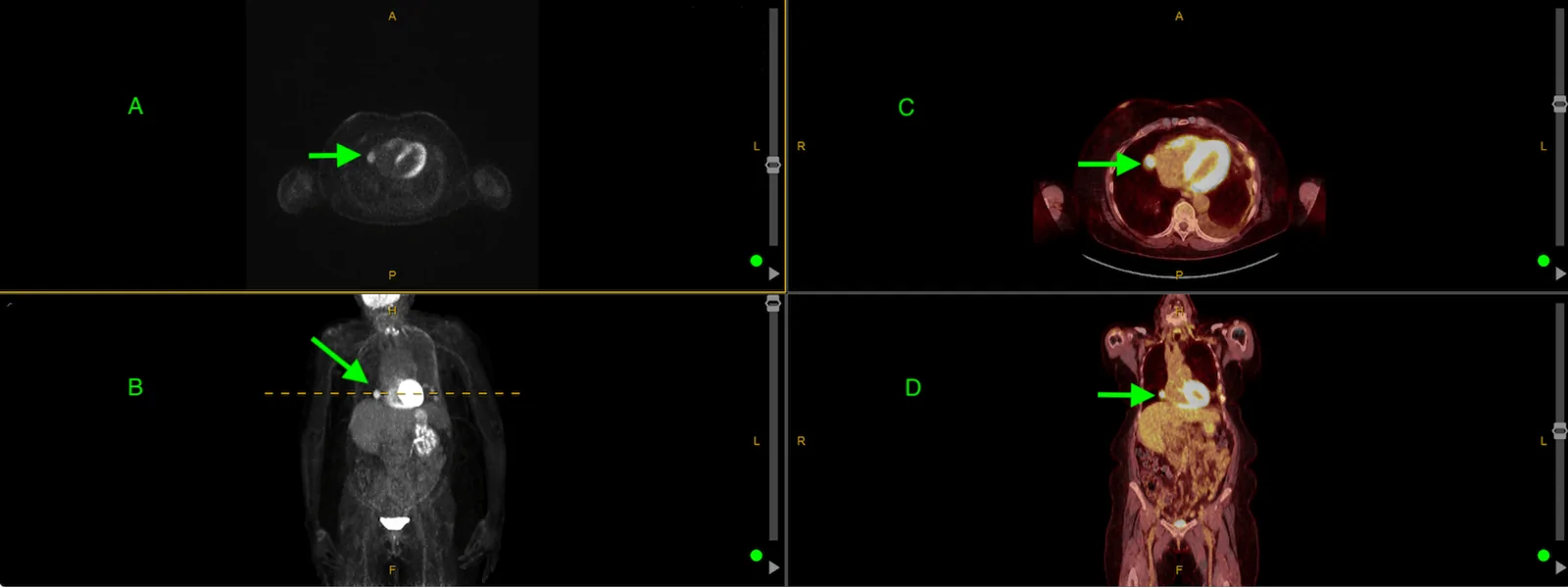
Panels A, B, C, and D show positron emission tomography (PET) scans demonstrating a solid hypermetabolic mass at the mediastinal border of the right middle lobe adjacent to the right atrium (arrow points to the mass), with a small left pleural effusion (A: PET axial view, B: PET coronal view, C: computerized tomography (CT)-fused axial view, D: CT-fused coronal view).

Pulmonary function testing showed a severe (stage III) obstructive pattern with a significant bronchodilator response and mildly reduced lung volumes, consistent with COPD (Table 2).

**Table 2:** Pulmonary function test Findings indicate a severe obstructive pattern with significant bronchodilator response (FEV1 + 230 mL) and air trapping, with mildly reduced lung volumes. BD: bronchodilator; FEV1: forced expiratory volume in 1 second; FVC: forced vital capacity; TLC: total lung capacity; RV: residual volume

Parameter	Pre-BD	Post-BD	% Predicted
FEV1 (L)	1.41	1.64	52
FVC (L)	2.13	2.33	57
FEV1/FVC (%)	66	70	-
TLC (L)	5.03	-	76
RV (L)	2.73	-	117
RV/TLC (%)	54	-	-

Diagnosis, management, and clinical course

Initial Diagnosis and Management

The constellation of recurrent dependent purpura, systemic inflammation, positive serum cryoglobulins with a type III immunofixation pattern, low-normal C4, a negative ANA panel, and chronic HCV established HCV-associated type III mixed cryoglobulinemic vasculitis with cutaneous and probable renal involvement. The patient’s cocaine use mandated consideration of levamisole-adulterated cocaine vasculopathy as a mimic. After symptomatic improvement with 100-40 mg glecaprevir/pibrentasvir (Mavyret) and a five-day course of 40 mg prednisone tablets, she was started on 20 mg prednisone tablets for 10 days (with a planned taper) and referred to rheumatology, with HCV-directed antiviral therapy as the cornerstone of treatment for the underlying driver.

Rheumatology Follow-Up

Rheumatology co-management spanned two visits. At the first visit, approximately six weeks into antiviral therapy, the diagnosis was confirmed clinically and serologically. The left lower extremity bore the predominant disease burden: circumferential erythema, pitting edema, and tenderness to palpation, with bilateral petechiae of the legs and dorsal forearm ecchymoses. Creatinine was normal; urine studies and CRP were ordered to monitor for renal involvement. Prednisone was continued at 10 mg daily as a bridge to antiviral effect, 40 mg pantoprazole tablets daily were added, and anti-human neutrophil elastase antibody (anti-HNE antibody) testing was considered to evaluate concurrent levamisole vasculopathy. At the second visit, the patient had completed the two-month antiviral regimen with 100-40 mg glecaprevir/pibrentasvir (Mavyret) three tablets daily with lunch, and HCV RNA was undetectable, confirming sustained virologic response. Cutaneous disease had resolved: no purpura or ulcerations on examination, with only residual distal left leg hyperpigmentation markedly improved from baseline. Urine protein-to-creatinine ratio was unremarkable, excluding active glomerulonephritis. Prednisone was tapered to 5 mg daily, where it remains with ongoing taper. For the pulmonary mass, thoracentesis cytology was negative, and the patient was referred to cardiothoracic surgery for biopsy. Tobacco use had decreased from one pack to one to five cigarettes daily; cocaine abstinence was maintained. A summary of the clinical course is shown in Table 3.

**Table 3:** Clinical course timeline LE: lower extremity; HCV: hepatitis C virus; CMP: comprehensive metabolic panel; PT/INR: prothrombin time/international normalized ratio; aPTT: activated partial thromboplastin time; CT: computed tomography; PET-CT: positron emission tomography-computed tomography; C3/C4: complement components 3 and 4; CRP: C-reactive protein; anti-HNE: anti-human neutrophil elastase antibody

Visit	Key findings	Management	Response/status
Initial presentation	Recurrent lower-extremity (LE) rash with macular-papular eruption, pruritus, and edema	10-day course of cephalexin, ibuprofen (200 mg), and acetaminophen (325 mg) as needed	No improvement during antibiotic course; rash spontaneously resolved after several months
Six-month follow-up	Bilateral LE pain, forearm abrasions and bruising, recurrent rash and bruising in all extremities	Topical mupirocin 2%; CMP, PT/INR, aPTT, and hepatitis panel obtained to evaluate liver function	Hepatitis C virus RNA detected; informed the patient's gastroenterologist for treatment; genotyping confirmed HCV genotype 3 with METAVIR stage F2; glecaprevir-pibrentasvir (Mavyret) 100/40 mg initiated
Six-week follow-up	Recurrent LE purpura, bilateral bruising and edema, severe LE tenderness, HCV-positive, reported cocaine use	Prednisone (40 mg) PO × five days, oxycodone (5 mg) twice daily as needed × five days, CT angiography of the aorta with bilateral iliofemoral runoff, urinalysis, protein/creatinine ratio, cryoglobulin, C3, C4 ordered	Right middle lobe mass identified on CT, symptoms improved with prednisone (40 mg), cryoglobulin elevated
Two-week follow-up	Recurrent LE purpura, positive cryoglobulins (type III), hematuria/proteinuria, right middle lobe mass, HCV therapy ≈ 8 weeks underway	Prednisone (20 mg) daily, rheumatology referral, dedicated chest CT followed by PET-CT	Type III cryoglobulinemia diagnosis established, symptoms improved on prednisone (20 mg) and glecaprevir-pibrentasvir 100/40 mg, pulmonology referral placed
Rheumatology visit, one month later	LE circumferential erythema with pitting edema, bilateral petechiae, creatinine normal	Prednisone continued at 10 mg daily, proton-pump inhibitor prescribed, urine studies and CRP ordered, anti-HNE antibody considered, cocaine abstinence reinforced	Disease is clinically active but stable, renal function preserved
Two-month rheumatology follow-up	HCV RNA undetectable (sustained virologic response), rash resolved, LE swelling cleared, urine protein/creatinine ratio unremarkable, residual LE hyperpigmentation	Pulmonary mass evaluation ongoing; thoracentesis performed (cytology negative), CT-surgery referral for biopsy, tobacco cessation counseled	Cutaneous vasculitis in remission, no active glomerulonephritis, prednisone taper in progress

## Discussion

This case illustrates a common and consequential pitfall: recurrent, dependent, antibiotic-unresponsive, lower-limb purpura misattributed to cellulitis. Cryoglobulinemic vasculitis arises when cold-precipitable immunoglobulins deposit in small vessels and activate complement, producing a leukocytoclastic small-vessel vasculitis [1,2]. In HCV infection, chronic B-cell stimulation drives the production of rheumatoid factor-containing immune complexes, and HCV is identified in the great majority of patients with mixed cryoglobulinemia, supporting a causal relationship [3,4]. Hepatitis C and mixed cryoglobulinemia synergistically decrease C4 levels, and C4 is disproportionately lowered relative to C3 in patients with immune complex-mediated disease as opposed to other disorders that consume complement [3,8]. 

Type III mixed cryoglobulinemia comprises only polyclonal immunoglobulins and is often associated with a lower cryoglobulin fraction. It may represent an earlier immunologic state along the spectrum toward type II, but the clinical features of the two mixed forms overlap [1,3]. The prototypical presentation is the Meltzer triad of purpura, weakness, and arthralgia, with cutaneous disease most common and renal (typically membranoproliferative glomerulonephritis) and peripheral nerve involvement representing major sources of morbidity [2,3,6]. Our patient demonstrated cutaneous disease together with hematuria and low-grade proteinuria at presentation, signaling renal involvement; however, repeated urine protein-to-creatinine ratio measurement after initiation of antiviral therapy was unremarkable, and active glomerulonephritis was not confirmed on follow-up. This evolution is consistent with the characteristic pattern of a depressed C4 with relatively preserved C3 (here C4 at the low end of normal) and underscores the value of serial renal monitoring rather than proceeding directly to immunosuppression when renal function is preserved [2,3].

Several diagnostic caveats deserve emphasis. Cryoglobulin testing is prone to false-negative results unless the sample is collected and transported at 37°C and allowed to clot while warm; so, repeated negative results may reflect pre-analytic handling rather than true absence [6]. Definitive confirmation may include a skin biopsy demonstrating leukocytoclastic vasculitis and, where renal involvement is suspected, a renal biopsy. Neither was performed in this patient, and the diagnosis rested on the combination of clinical findings and serology. Recurrence and failure to respond to antibiotics, as occurred here for more than a year, should prompt reconsideration of cellulitis and a vasculitis workup. The patient’s easy bruising and skin fragility broadened the differential to include nutritional causes, such as vitamin C deficiency, but the recurrent, inflammatory, purpuric picture with positive cryoglobulins pointed to vasculitis.

A key mimic in this patient was levamisole-adulterated cocaine vasculopathy, given her cocaine use. That entity classically produces retiform purpura favoring the ears, cheeks, and extremities, with neutropenia, high-titer antineutrophil cytoplasmic antibodies (frequently both myeloperoxidase and proteinase-3 specificities, plus anti-human neutrophil elastase), and a relative paucity of true autoimmune end-organ damage; lesions often improve with drug cessation [9]. Features favoring cryoglobulinemic vasculitis in our patient included positive cryoglobulins with a type III immunofixation pattern, chronic HCV as a defined driver, and the absence of the pathognomonic retiform or auricular lesions. Rheumatology considered anti-HNE antibody testing at the first follow-up visit to further evaluate levamisole vasculopathy as a concurrent contributor; when the two diagnoses overlap, antineutrophil cytoplasmic antibody testing and skin biopsy help to separate them and are advisable. The patient’s sustained cocaine abstinence after bronchoscopy cancellation removed an ongoing confound and supported clinical interpretation of her improving course.

Management and treatment response

Management of cryoglobulinemic vasculitis targets the underlying cause. HCV-directed direct-acting antivirals achieve high rates of sustained virologic response and can induce remission of vasculitis, with published data indicating cutaneous improvement in 60-90% of patients following viral eradication [3,4]. Our patient achieved undetectable HCV RNA after a two-month 100-40 mg Mavyret (three tablets daily) antiviral regimen, with complete resolution of cutaneous vasculitis on clinical examination. This outcome directly supports antiviral cure as the cornerstone of treatment and illustrates the principle that immunosuppression alone, without addressing the underlying driver, provides only temporary benefit. Corticosteroids served as a bridge to antiviral effect: prednisone was initiated with a 40 mg course daily for five days, transitioned to 20 mg daily, continued at 10 mg at rheumatology follow-up, and successfully tapered to 5 mg daily after viral cure was confirmed; the patient remains on this dose with ongoing taper. Active or severe vasculitis, particularly with significant renal involvement, may require B-cell depletion with rituximab, but the normalization of urine studies in this patient reduced the urgency for escalated immunosuppression [3,4].

Tobacco use represents an additional modifiable vascular risk factor. Smoking promotes endothelial dysfunction and compounds disease burden in small-vessel vasculitis. At the second rheumatology visit, tobacco cessation was counseled, and the patient had meaningfully reduced her smoking from approximately one pack per day to one to five cigarettes daily. Continued reduction to complete cessation remains an important therapeutic goal.

The incidentally discovered hypermetabolic pulmonary and pleural mass in this heavy smoker is concerning for malignancy and warrants tissue diagnosis. Although malignancy is itself an uncommon secondary cause of mixed cryoglobulinemia, HCV remained the established driver in this patient. Thoracentesis yielded pleural fluid with negative cytology, and cardiothoracic surgery referral for definitive biopsy has been made. The mass is best regarded as a separate, important finding requiring independent evaluation.

Limitations

Limitations include the single-patient design, the absence of confirmatory skin or renal biopsy, limited cryoglobulin quantification, incomplete documentation of antineutrophil cytoplasmic antibody and anti-HNE antibody status to fully exclude concurrent levamisole vasculopathy, and incomplete follow-up on the pulmonary mass. The ongoing corticosteroid taper and evolving pulmonary workup preclude a definitive long-term outcome assessment at this time.

## Conclusions

Recurrent, antibiotic-unresponsive, dependent lower-extremity purpura should raise suspicion for cutaneous small-vessel vasculitis, and in patients with chronic HCV, for cryoglobulinemic vasculitis in particular. A focused workup with serum cryoglobulins with appropriate warm-handling, complement (notably C4), urinalysis for renal involvement, antinuclear and antineutrophil cytoplasmic antibodies to address mimics, and HCV testing can establish the diagnosis and redirect care from repeated antibiotics toward antiviral and, when indicated, immunosuppressive therapy. In patients who use cocaine, levamisole-adulterated cocaine vasculopathy is an important differential, and anti-HNE antibody testing should be considered.

In this patient, HCV eradication with direct-acting antivirals led to complete resolution of cutaneous vasculitis, normalization of serial urinalysis without evidence of active glomerulonephritis, and successful corticosteroid tapering. The patient remains on 5 mg prednisone daily with ongoing taper toward discontinuation. Early rheumatologic co-management facilitated systematic monitoring for renal involvement, titration of corticosteroids as a bridge to antiviral effect, and structured evaluation of levamisole-adulterated cocaine vasculopathy as a concurrent mimic. This case reinforces antiviral cure as the primary treatment target in HCV-associated cryoglobulinemic vasculitis and highlights the importance of early diagnosis in shortening diagnostic delay and redirecting therapy toward the underlying driver.
